# Dual Role of Glutathione as a Reducing Agent and Cu-Ligand Governs the ROS Production by Anticancer Cu-Thiosemicarbazone Complexes

**DOI:** 10.1021/acs.inorgchem.2c04392

**Published:** 2023-02-20

**Authors:** Alessandra G. Ritacca, Enrico Falcone, Iman Doumi, Bertrand Vileno, Peter Faller, Emilia Sicilia

**Affiliations:** † Department of Chemistry and Chemical Technologies, Università della Calabria, Ponte P. Bucci, 87036 Arcavacata di Rende (CS), Italy; ‡ Institut de Chimie (UMR 7177), University of Strasbourg − CNRS, 4 Rue Blaise Pascal, 67000 Strasbourg, France; § Institut Universitaire de France (IUF), 1 rue Descartes, 75231 Paris, France

## Abstract

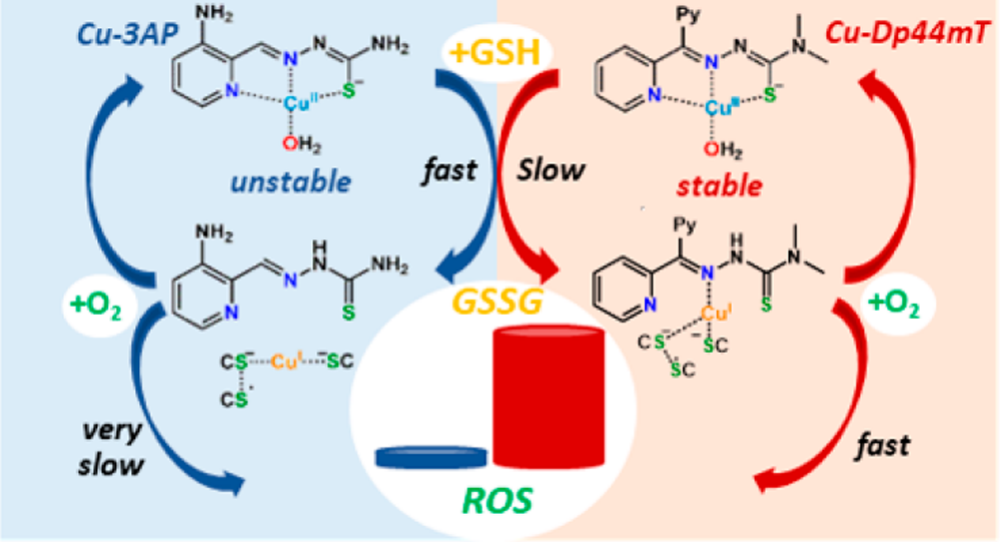

α-Pyridyl thiosemicarbazones (TSC) such as Triapine (3AP) and Dp44mT are a promising class of anticancer agents. Contrary to Triapine, Dp44mT showed a pronounced synergism with Cu^II^, which may be due to the generation of reactive oxygen species (ROS) by Dp44mT-bound Cu^II^ ions. However, in the intracellular environment, Cu^II^ complexes have to cope with glutathione (GSH), a relevant Cu^II^ reductant and Cu^I^-chelator. Here, aiming at rationalizing the different biological activity of Triapine and Dp44mT, we first evaluated the ROS production by their Cu^II^-complexes in the presence of GSH, showing that Cu^II^-Dp44mT is a better catalyst than Cu^II^-3AP. Furthermore, we performed density functional theory (DFT) calculations, which suggest that a different hard/soft character of the complexes could account for their different reactivity with GSH.

## Abstract 2

The catalytic activities of two Cu^II^ complexes of the anticancer thiosemicarbazones 3AP and Dp44mT in the presence of GSH for ROS production have been compared. Cu^II^-3AP is reduced fast by GSH, and the following detachment of the ligand slows down ROS production. Cu^II^-Dp44mT is reduced slowly, but formation of the stable ternary complex Dp44mT-Cu^I^-GS speeds up ROS production. Due to its stability, the complex Cu^II^-Dp44mT has higher biological activity than Cu^II^-3AP.

## Introduction

**Scheme 1:**
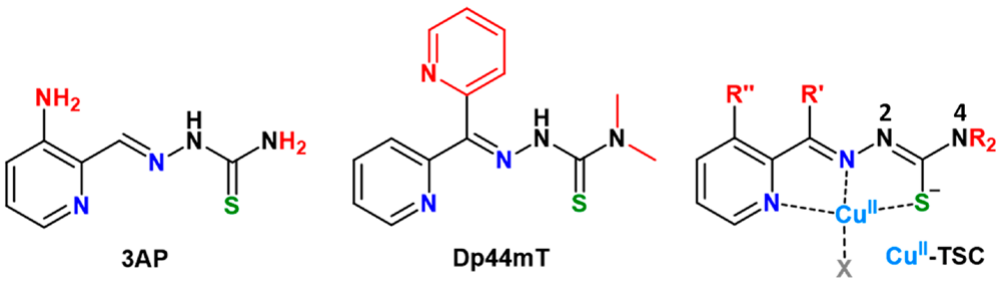
Structures of 3AP, Dp44mT, and Their Cu^II^-TSC Complex Cu-binding atoms are colored in blue/green and structural differences are highlighted in red.

Cancer cells show increased Cu levels, which are required to sustain cell proliferation, angiogenesis and metastasis.^1^ Accordingly, Cu-activated (pro)drugs are gaining increasing interest because they could potentially enhance the selectivity toward cancer vs healthy cells.^2−4^ However, the intracellular stability of Cu-complexes is challenged by endogenous Cu-binding molecules, such as glutathione (GSH) and metallothioneins.^5^ Among the developed metal-based anticancer agents, α-pyridyl thiosemicarbazones (TSCs) are promising candidates. These are tridentate ligands that form metal complexes upon deprotonation of the thioamide group (see Scheme 1).^6^ Triapine (3-aminopyridine-2-carboxaldehyde thiosemicarbazone, 3AP, see Scheme 1) was the first TSC representative undergoing more than 30 clinical trials as an anticancer agent, which finally failed because of some adverse effects. Later, the di-2-pyridylketone TSC Dp44mT (di-2-pyridylketone 4,4-dimethyl-3-thiosemicarbazone, see Scheme 1) and its derivative DpC (di-2-pyridylketone 4-cyclohexyl-4-methyl-3-thiosemicarbazone), which is currently implicated in clinical trials (NCT02688101), showed higher cytotoxicity (nM range) than 3AP (μM range) and more advantageous pharmacokinetics. The best-known mode of action of anticancer TSCs is iron chelation and the inhibition of the Fe-dependent enzyme ribonucleotide reductase, which is essential for DNA synthesis and cell proliferation, was the first identified target. Since then multiple other effects have been described.^6−9^

In addition to iron, Cu chelation also emerged as an additional mechanism for some TSCs. In particular, Dp44mT and its derivatives, but not 3AP, have shown a pronounced synergism with Cu^II^ salts, suggesting the involvement of Cu chelation and, possibly, Cu-catalyzed production of reactive oxygen species (ROS) in their mode of action. Indeed, Cu^II^-Dp44mT was shown to produce ROS in the presence of cysteine *in vitro* and to reduce the GSH/GSSG ratio in cells, whereas weak ROS generation was observed for Cu^II^-3AP in intracellular conditions.^9−11^ This has been assigned to the catalytic activity of Cu^II^-TSC in the reaction of GSH with O_2_ to form ROS and GSSG, in which Cu cycles between Cu^II^ and Cu^I^. In this respect, it is noteworthy that the two 5-membered chelate rings formed by the (N_py_, N, S^–^) donors, as shown in Scheme 1, disfavor Cu^I^ binding, resulting in a negative reduction potential (ca. −0.2 V).^8,9^

Of note, several structure–activity studies have shown that the dimethylation of the terminal N^4^ nitrogen (see Scheme 1) enhances the affinity for Cu^II^ and the cytotoxicity. It is worth mentioning that Dp44mT has approximately a 100-fold higher Cu^II^-affinity than 3AP.^12−14^ Moreover, a correlation has been recently found between the cytotoxicity of several TSCs, including 3AP and Dp44mT and the reduction rate of their Cu^II^ complexes by the intracellular reductant GSH.^14^ Notably, in anaerobic conditions, physiologically relevant concentrations of GSH reduced Cu^II^-3AP much faster than Cu^II^-Dp44mT. In the presence of O_2_, Cu^II^-3AP undergoes reduction by GSH and partial dissociation, whereas Cu^II^-Dp44mT forms a kinetically stable yet redox-active GS^–^-Cu^II^-Dp44mT complex.^14−16^ However, a direct comparison of the ROS production by these two Cu-TSC complexes has never been reported. Since we recently showed that the reduction of Cu^II^ is the rate-limiting step of the ROS production by Cu-TSC complexes,^16^ Cu^II^-3AP, in spite of its lower cytotoxicity, is predicted to produce ROS faster than Cu^II^-Dp44mT. Here, we assessed the ROS generation and GSH oxidation by these two Cu^II^-TSC complexes showing, instead, that Cu^II^-Dp44mT produces ROS faster than Cu^II^-3AP does. Indeed, the partial dissociation of Cu^II^-3AP by GSH, forming poorly redox-active Cu-GS clusters, undermines its ability to produce ROS despite its faster reduction. Thus, the higher cytotoxicity of Cu^II^-Dp44mT compared to Cu^II^-3AP can be explained by their different fate in the presence of GSH.

Besides, the rationale behind the difference in reactivity of these complexes with GSH is unclear. Noteworthy, although the above-mentioned higher affinity of Dp44mT for Cu^II^ can contribute, it is not exhaustive, as the competition between the TSC and GSH is substantially played at the reduced Cu^I^ level. In order to shed light on the different behavior between the two Cu^II^-TCS complexes with GSH, we carried out a quantum mechanical DFT exploration of the Cu^II^-3AP/GSH system, which was compared to our previous computational studies of the reactivity of the Cu^II^-Dp44mT complex. The different hard/soft character of the two ligands and their complexes appeared as a possible explanation of the observed reactivity differences.

## Results

### ROS Production by Cu^II^-TSCs Complexes in the Presence of GSH

**Figure 1:**
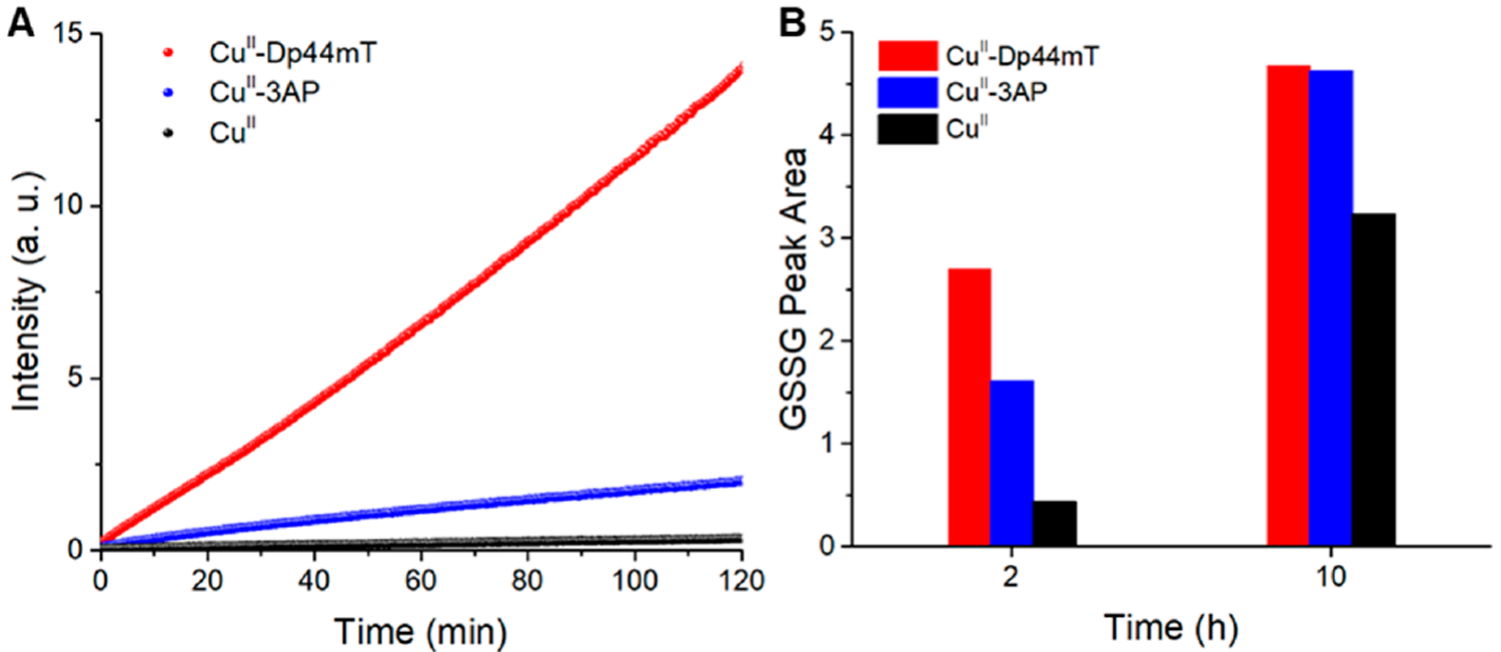
ROS (A) and GSSG (B) formation by Cu^II^-Dp44mT (red), Cu^II^-3AP (blue), and Cu^II^ (black) in the presence of O_2_ and GSH. Conditions: [Cu^II^] = 27 μM, [TSC] = 30 μM, [GSH] = 3 mM; [H_2_DCF] = 10 μM, [H_2_O_2_] = 500 μM (A); HEPES buffer 100 mM pH 7.4.

First, we measured the formation of ROS catalyzed by Cu^II^-TSC complexes and “free” Cu^II^ by means of the fluorometric DCF (dichlorofluorescein) assay (see Figure 1A).^17^ Interestingly, both complexes showed faster ROS production than “free” Cu^II^. Moreover, Cu^II^-Dp44mT induced a much faster ROS production than Cu^II^-3AP, as also shown by EPR spin scavenging experiments using the nitroxyl radical TEMPOL (4-hydroxy-2,2,6,6-tetramethylpiperidin-1-oxyl) as a HO^•^ scavenger as shown in Figure S1 of the Supporting Information (SI). The same trend of reactivity was observed by monitoring the corresponding GSSG formation via HPLC (see Figure 1B). Considering that the reduction seems to be the rate limiting step in Cu^II^-Dp44mT in ROS production, and this step is slower for Cu^II^-Dp44mT compared to Cu^II^-3AP, these findings could seem counterintuitive.^14,16^ In other words, a higher activity of Cu^II^-3AP compared to Cu^II^-Dp44mT could be expected.

**Figure 2:**
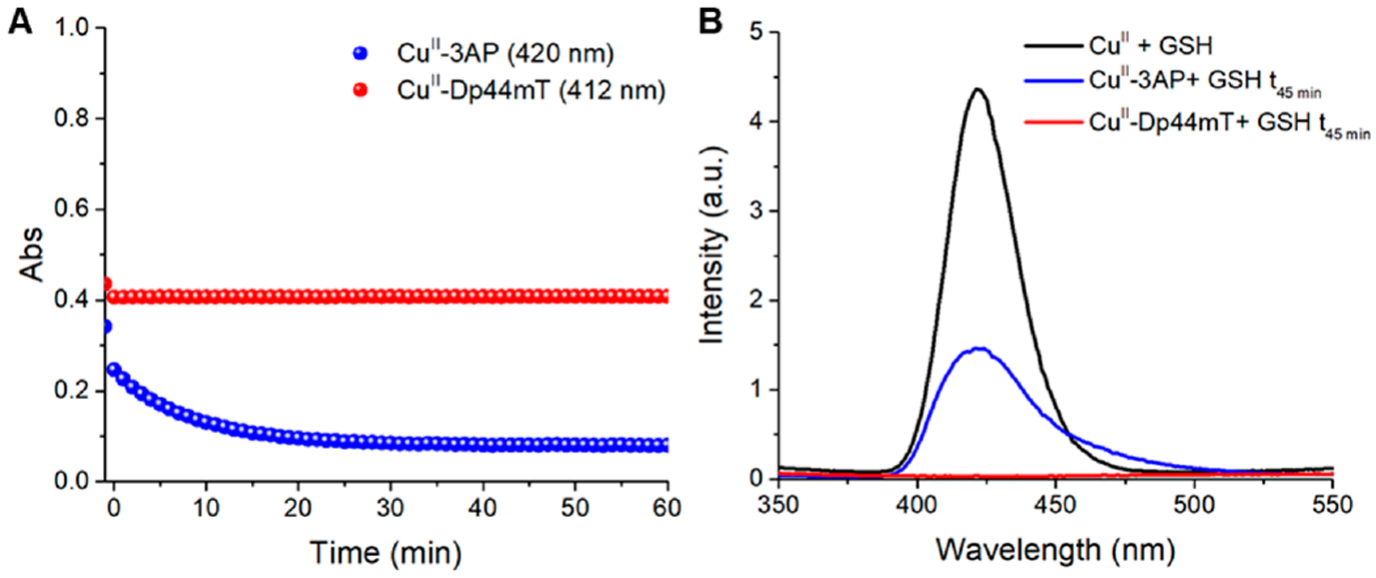
Stability of Cu^II^-Dp44mT (red) and Cu^II^ -3AP (blue) against GSH. (A) Absorbance of the characteristic charge transfer S → Cu^II^ bands of the Cu^II^-TSCs complexes; (B) low-temperature (77 K) luminescence spectra of Cu^II^-GSH (black) and Cu^II^-TSCs complexes after 45 min incubation with GSH. Conditions: [Cu^II^] = 27 μM, [TSC] = 30 μM, [GSH] = 3 mM, HEPES buffer 100 mM pH 7.4.

However, Cu^II^-3AP, unlike Cu^II^-Dp44mT, is also mostly (∼70%) dissociated upon reduction by GSH (see Figure 2A). Here, by means of low temperature (77 K) luminescence measurements (see Figure 2B), we proved that the Cu dissociated from 3AP was bound to GSH in Cu^I^_4_(GS)_6_ clusters with characteristic luminescence emission at 422 nm.^18^ Instead, no Cu^I^_4_(GS)_6_ was formed upon incubation of Cu^II^-Dp44mT with GSH.

**Figure 3:**
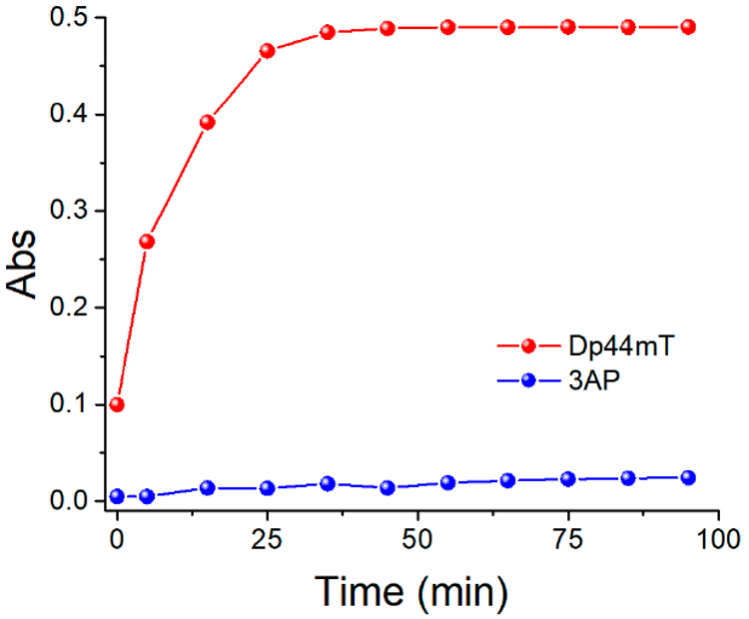
Kinetics of O_2_-induced Cu^I^_*n*_(GS)_*m*_ oxidation to (GS^–^)-Cu^II^-Dp44mT (red dots, 415 nm) or Cu^II^-3AP (blue dots, 420 nm). Conditions: [GSH] = 3 mM, [Cu^I^(MeCN)_4_PF_6_] = 27 μM, [TSC] = 30 μM, HEPES 100 mM pH 7.4 (DMSO, MeCN < 1%).

Besides, we assessed the reoxidation rate of GS-bound Cu^I^ in the presence of Dp44mT and 3AP. For this purpose, solutions of GSH, [Cu^I^(CH_3_CN)_4_]PF_6_, and Dp44mT or 3AP were thoroughly degassed under N_2_ and mixed into a sealable cuvette. Upon exposure to air, UV–vis spectra were recorded over time (see Figure 3). Remarkably, in the presence of Dp44mT, GS-bound Cu^I^ was readily oxidized to the ternary (GS^–^)-Cu^II^-Dp44mT upon air exposure, as shown by the appearance of charge transfer S(Dp44mT) → Cu^II^ band at 415 nm (red-shifted compared to the band of Cu^II^-Dp44mT in the absence of GSH) and S(GS) → Cu^II^ band at 327 nm (see Figure S2).^15,16^ Instead, oxidation to (GS^–^)-Cu^II^-3AP appeared to be negligible in the same time frame.

On balance, the formation of poorly redox active Cu^I^_4_(GS)_6_ species upon Cu^II^-3AP reduction can account for its lower reactivity compared to Cu^II^-Dp44mT. The residual portion of Cu^II^-3AP (∼30%) could be then responsible for its higher activity compared to “free” Cu^II^. Importantly, this behavior parallels the higher cytotoxicity of Cu^II^-Dp44mT compared to Cu^II^-3AP against cancer cells in culture, indicating that GSH depletion and GSH-mediated ROS production could be implicated in the cytotoxic mechanism.

### DFT Analysis of Cu^II^-3AP Reactivity with Glutathione

**Scheme 2:**
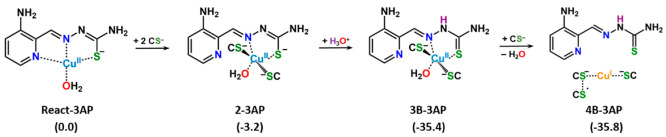
Calculated Steps of the Process Leading to the Reduction of the Cu^II^-3AP Complex in the Presence of Thiol Containing Molecules Relative energies are in kcal/mol and calculated with respect to the energy of separated reactants.

By means of DFT calculations, we previously described the mechanism of the reaction between Cu^II^-Dp44mT and GSH.^16^ Here, to understand the difference between Cu^II^-3AP and Cu^II^-Dp44mT in their reactivity with GSH, the reaction steps leading to the formation of the products of the GSH oxidation by the Cu^II^-3AP complex were examined starting from the Cu^II^-3AP complex in its prevalent protonation state (i.e., with the deprotonated hydrazinic N^2^, see Scheme 2). A water molecule occupies the forth position of the almost square planar geometry. l-Cysteine was used as a model to contain the computational cost and allow inclusion of an adequate number of reacting thiol-containing molecules. These same starting conditions were used for the Cu^II^-Dp44mT and hence allow direct comparisons.

The outcomes of the DFT exploration describing the main steps of the reaction mechanism are summarized in Scheme 2. Relative Gibbs free energies (Δ*G*^298 K^) of the intercepted stationary points were calculated with respect to the sum of the energies of the starting Cu^II^-(3AP)(H_2_O) complex, three deprotonated cysteines, Cys^–^, and a H_3_O^+^ molecule as the reference zero energy of the system. Fully optimized geometries of all the intermediates intercepted along the reaction pathway can be found in Figure S3 of the SI. For the sake of comparison, the analogous steps of the reduction reaction of the Cu^II^-Dp44mT complex are reported in Figure S4 of the SI.

**Figure 4:**
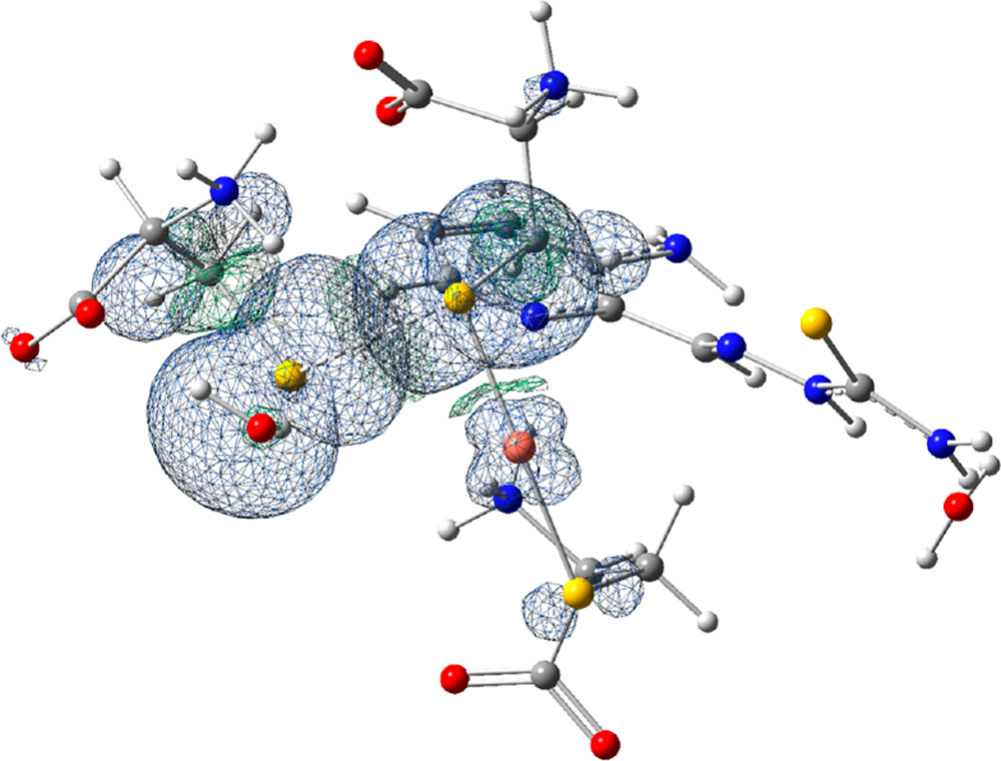
Spin density representation for the **4B-3AP** product of the reduction reaction.

The initial interaction of the Cu^II^-3AP complex with a Cys^–^ unit leads to the formation of a noncovalent adduct, named **1-3AP**, stabilized by an electrostatic interaction established between one of the water hydrogens and the approaching cysteine sulfur (see Figure S2S3). Formation of **1-3AP** is calculated to be exergonic by 7.2 kcal/mol with respect to the zero reference energy of separated reactants. When two additional Cys^–^ units are taken into consideration, a rearrangement occurs and a new intermediate, **2-3AP**, having a pseudo square-pyramidal geometry is formed (see Scheme 2 and Figure S3). The Cu^II^ center is coordinated to two deprotonated cysteines, one water molecule and the TSC (N, S^–^) set, whereas the N_py_ nitrogen is definitively detached. Such rearrangement, causing a slight destabilization of 4.0 kcal/mol, does not allow any further reorganization in the coordination sphere of the metal. Indeed, all the strategies deployed to intercept minima or transition states leading to either the detachment of the 3AP ligand or the reduction of the Cu^II^ center failed. Only by manually elongating the distance between copper and the bidentately bound 3AP ligand, it was possible to locate a fictitious minimum, **3A-3AP**, lying 7.3 kcal/mol (see Figure S3) above the reference zero energy limit, in which Cu appears to be reduced. Therefore, in analogy with the pathway described for the Cu^II^-Dp44mT complex involving the reprotonation of the hydrazinic N^2^, that appears to be the necessary step for the copper reduction to occur, also in this case ligand reprotonation was considered. The addition of a H_3_O^+^ ion, simulating a protonating agent, in close proximity of the **2-3AP** intermediate from the side of the ligand causes a proton shift on the hydrazinic nitrogen. The new formed **3B-3AP** intermediate is calculated to be more stable than the separated reactants by 35.4 kcal/mol. This intermediate spontaneously evolves into the final Cu^I^ product, **4B-3AP**, due to the reducing action of one of the Cys^–^, which causes the definitive detachment of both water and the 3AP ligand. The Cu^I^ center coordinated to two Cys^–^ adopts a linear geometry and the adduct is only slightly, by 0.4 kcal/mol, more stable than the preceding minimum. As seen in the case of Cu^II^-Dp44mT,^16^ from the geometrical arrangement of the deprotonated cysteines around the reduced copper, it is possible to draw the conclusion that one electron flows from the unbound cysteine to copper through a bridge formed with one of the cysteines coordinated to the metal, forming a disulfide radical anion (the calculated spin density of **4B-3AP** product is depicted in Figure 4).

Thus, the outcome of the DFT analysis is in agreement with the experimental findings showing the reduction and dissociation of Cu^II^-3AP by GSH.

### Comparison between 3AP and Dp44mT

The comparison between the reaction pathways of Cu^II^-3AP and Cu^II^-Dp44mT (see Figures S3 and S4) reveals that, once the hydrazinic nitrogen is reprotonated, the reduction occurs very quickly for the 3AP complex, without the involvement of a transition state, which is instead observed in the reduction of Cu^II^-Dp44mT. The absence of a transition state and the reduced number of steps required for the reduction to occur are in line with the experimentally observed faster reduction of Cu^II^-3AP compared to Cu^II^-Dp44mT by GSH under anaerobic conditions.^14^ Moreover, very importantly, unlike Dp44mT, 3AP ligand is completely detached. Interestingly, the reprotonation of the hydrazinic nitrogen is required in both complexes to loosen the bond between Cu and the TSC sulfur and favor the reduction.

In order to find a possible explanation of this difference in behavior, we focused our attention on the difference in hard/soft character of the two ligands and their copper complexes and invoked the HSAB principle.^19,20^ The binding of water to the metal center in the Cu^II^-3AP complex and, on the contrary, its detachment and substitution with a Cys^–^ unit in the Cu^II^-Dp44mT are an indication of the softer character of the copper center and, in turn, of the ligand, in the latter case. Also the difficulty of the Cu center in the Cu-Dp44mT complex to break the bond with the S donor atom of the ligand clearly indicates a softer character of the system.^16^ As a consequence, the reduction of the Cu^II^-3AP complex is accompanied by the Cu^I^ transfer from the harder ligand to the softer cysteines. It is also noteworthy that the presence of water in the equatorial plane of the **2-3AP** intermediate constrains the two Cys^–^ to bind axially, favoring the formation of the linear Cys-Cu^I^–Cys product. In the complex with the softer Dp44mT ligand, instead, Cu^II^ reduction does not imply the detachment from the ligand. Indeed, the bond with the N atom of the ligand does not break, and a ternary Cu^I^ species is observed.

**Figure 5:**
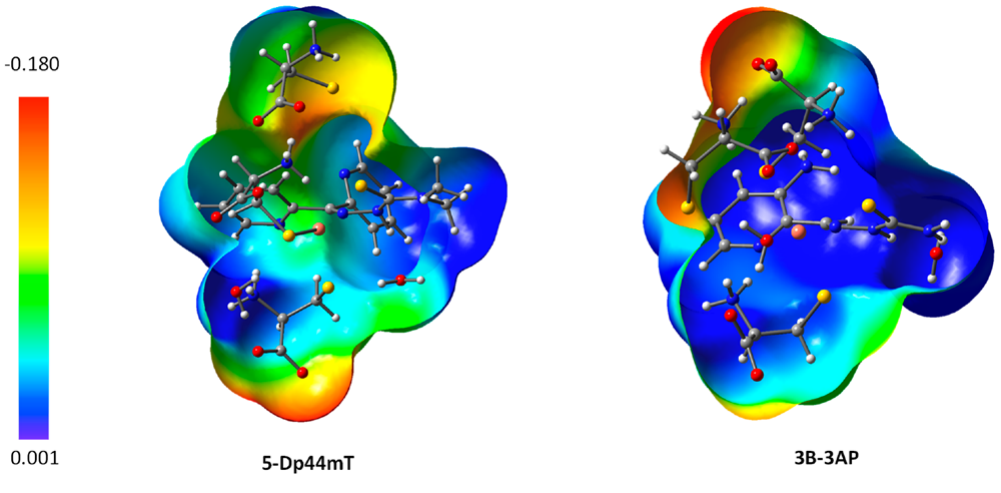
Maps of the molecular electrostatic potential MEP) for 3B-3AP and 5-Dp44mT intermediates, intercepted along the two compared reduction pathways, immediately after protonation of the hydrazinic nitrogen. The electrostatic potential is represented with a color scale going from red (−0.018 au) to blue (0.001 au). Red color is used to indicate regions with electron charge excess, and blue color for regions of electron charge deficiency. Green color evidence regions with intermediate character.

This difference in hard/soft character of the two ligands can be corroborated by the values of the global hardness and polarizability together with the maps of the molecular electrostatic potential (MEP). The calculated values of the global hardness are 2.85 and 2.60 for 3AP and Dp44mT, respectively, whereas the polarizability values are 28.76 Å^3^ for 3AP and = 38.73 Å^3^ for Dp44mT, confirming the softer nature of the Dp44mT ligand. The different hard/soft character of the corresponding complexes is graphically depicted using the maps of MEP for the two minima, **3B-3AP** and **5-Dp44mT**, intercepted along the two compared pathways, immediately after protonation of the hydrazinic nitrogen (see Figure 5). Such maps illustrate the three-dimensional charge distributions of molecules, highlighting the area of electron excess and electron deficiency. As hard species have high charge states due electron charge depletion and are weakly polarizable, while soft species have instead low charge states due to electron charge accumulation and are strongly polarizable, the colors of the plotted MEPs confirm that in the region of the 3AP ligand there is a lower concentration of negative electron charge with respect to the region occupied by the Dp44mT ligand corroborating the accentuated soft character of the latter.

In our current analysis water was used as the fourth Cu ligand in the starting complex, an assumption that could be unlikely in a real biological medium, where many other ligands with higher affinity, such as imidazole (histidine side-chain), are present. Therefore, in order to avoid that our analysis could be biased by this choice, we also examined the reactivity of Imidazole-Cu-TSC ternary complexes with GSH. DFT calculations showed that the detachment of the Imidazole ligand from the Cu center does not take place spontaneously. For the release to occur, indeed, an energy barrier has to be surmounted for both imidazole-Cu-TSC complexes. This behavior is in line with the soft/hard character of the imidazole ligand that, being classified as a borderline base, has a higher affinity than water for the borderline Cu^II^ acid center. The corresponding intercepted minima and transition states, together with their relative energies, describing how the detachment of the imidazole ligand occurs for both imidazole-Cu-TSC complexes, are reported in Figure S5. The successive steps of the mechanism, leading to the reduction of the two complexes, are not influenced by the identity of the fourth Cu ligand. Consistently, by addition of imidazole or glycine 3 mM no significant effect on both Cu^II^-3AP reduction by GSH and GSSG formation from Cu^II^-Dp44mT was observed (see Figures S6 and S7). This is in agreement with recent studies on the reduction of the Cu^II^-GHK complex by GSH.^21^

Moreover, the calculated barrier for the detachment of the imidazole from Cu-3AP (3.9 kcal/mol) is lower than that calculated for the imidazole-Cu-Dp44mT complex (6.9 kcal/mol). This difference further confirms that the borderline character of the Cu^II^ center is modulated differently by the binding with the two TSC ligands. Notably, the lower barrier for the detachment of imidazole from Cu-3AP compared to Cu-Dp44mT further suggests a harder character of the former complex.

## Discussion

**Scheme 3:**
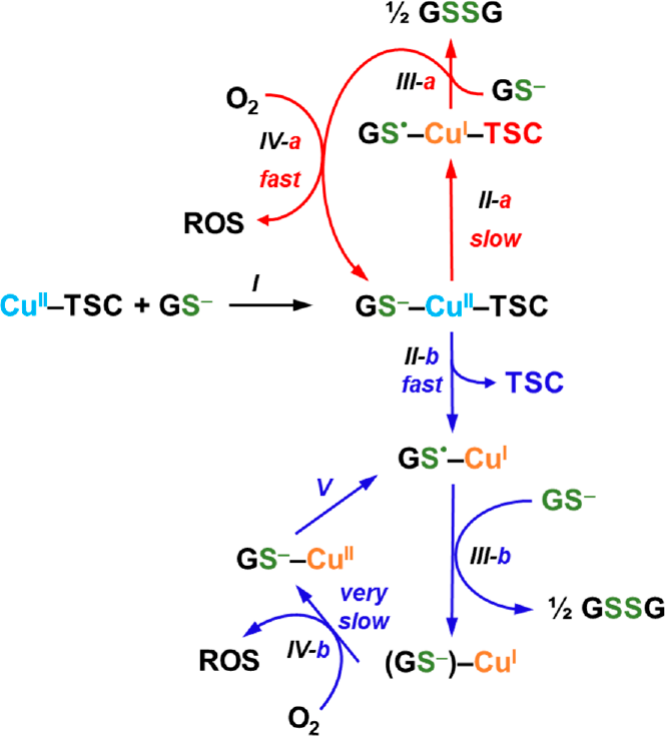
Different Fate of Cu^II^-TSCs in the Presence of GSH and Impact on the Generation of ROS Cu^II^-Dp44mT follows the red pathway: (*I*) formation of a ternary complex with GSH; (*II-a*) Cu reduction and formation of a ternary TSC-bound Cu^I^ intermediate; (*III-*a) formation of GSSG; (*IV-a*) re-oxidation to Cu^II^-Dp44mT by O_2_ with ROS production. Cu^II^-3AP follows mainly the blue pathway: (*II-b*) reduction to GS^•^-Cu^I^ and dissociation of the ligand; (*III-b*) formation of Cu^I^_*n*_(GS)_*m*_ clusters and GSSG; (*IV-b*) re-oxidation of Cu^I^_*n*_(GS)_*m*_ clusters to a putative GS^–^-Cu^II^ complex by O_2_ with ROS production; (*V*) reduction of GS^–^-Cu^II^ to GS^•^-Cu^I^.

The outcomes of our experimental and theoretical analysis of the reduction of Cu^II^-3AP and Cu^II^-Dp44mT by GSH and the experimentally evidenced ROS production are summarized in Scheme 3. First, a ternary complex is formed between Cu^II^-TSCs and GSH (step *I*). Then, for the reduction of the Cu^II^-TSC complexes, two different paths can be followed. Our findings suggest that Cu^II^-Dp44mT follows the path “*a*” (red), while Cu^II^-3AP *mostly* undertakes the path “*b*” (blue). Indeed, we showed that upon reduction the 3AP-bound copper is transferred to GSH (steps *II-b* and *III-b*). The latter stabilizes very much the Cu^I^ state into little redox-active Cu-thiolate clusters, implying a very slow Cu reoxidation and ROS production (steps *IV-b* and *V*). In contrast, for Cu^II^-Dp44mT, Cu^I^ stays partially coordinated to the TSC ligand and can be quickly reoxidized by O_2_ to yield ROS (step *IV-a*). Thus, despite the faster reduction of Cu^II^-3AP by GSH, which represents the rate-limiting step in the redox cycling, the overall ROS production is slower for this complex because it is less resistant against dissociation by GSH. Notwithstanding, Cu^II^-3AP oxidizes GSH and produces ROS faster than “free” Cu^II^, which can be explained considering that a minor portion of 3AP-bound Cu withstands GSH and undergoes the path “*a*”.

## Conclusions

In this study, we evaluated and compared the ROS production by the Cu^II^ complexes of the anticancer thiosemicarbazones 3AP and Dp44mT in the presence of GSH. This can be particularly relevant in cell compartments (e.g., the endoplasmic reticulum and mitochondria) or organisms (e.g., bacteria) devoid of metallothioneins, which also jeopardize the stability of Cu^II^-TSCs.^5,15^

Experiments showed that, although GSH reduced Cu^II^-3AP much faster than Cu^II^-Dp44mT, the latter produced ROS faster because, unlike 3AP, it withstands the reductive dissociation by GSH. Moreover, DFT investigations confirmed that reduction and dissociation spontaneously occur for the Cu^II^-3AP complex, while Dp44mT does not detach from Cu even upon reduction. Interestingly, simulations showed that in the Cu^II^-3AP complex Cu keeps its bond with water whereas the latter is replaced by a Cys^–^ unit in the Cu^II^-Dp44mT, suggesting a softer character of the latter complex, which could, hence, better compete against GSH. Furthermore, also the difficulty in breaking the bond with the S donor atom of the Dp44mT ligand corroborates the softer nature of the Cu^II^-Dp44mT complex. Of note, the different observed pro-oxidant activity may explain the Cu^II^ synergism and higher cytotoxicity of Dp44mT compared to 3AP and highlights once more the significance of GSH as a threatening factor for the stability and biological activity of Cu-complexes. Both calculations and experiments showed that the course of the process is not influenced by the identity of the fourth ligand bound to the Cu center in the starting complex.

## Materials and Methods

### Materials

All solvents and reagents obtained from commercial suppliers were used without further purification. TSCs were kindly provided by Dr. Christian R. Kowol (University of Vienna).

### Preparation of Stock Solutions

TSC stock solutions were prepared in DMSO, and their concentration was verified via spectrophotometric Cu^II^ titrations. Cu^II^ stock solution was prepared dissolving CuCl_2_·2H_2_O in ultrapure water (ρ = 18.2 MΩ·cm^–1^) and its concentration was verified by UV–vis spectroscopy (ε_780_ = 12 M^–1^cm^–1^). A stock solution of HEPES buffer (500 mM, pH 7.4) was prepared by dissolving free acid powder in ultrapure water and adjusting the pH with NaOH. A stock solution of phosphate buffer (PB, 500 mM, pH 7.4) was prepared by mixing KH_2_PO_4_ with K_2_HPO_4_ in ultrapure water and adjusting the pH with NaOH. GSH stock solutions were prepared in ultrapure water on a daily basis. A stock solution of H_2_DCFDA (5 mg/mL) was prepared in ethanol. A stock solution of [Cu^I^(CH_3_CN)_4_]PF_6_ was prepared in CH_3_CN. TEMPOL stock solution was prepared in ultrapure water.

### DCF Assay

H_2_DCF (2,7-dihydrodichlorofluorescein) was freshly prepared by alkaline hydrolysis of H_2_DCFDA (2,7-dihydrodichlorofluorescein diacetate) adapting a previously reported protocol: 100 μL of a 1 mM solution of H_2_DCFDA in ethanol was mixed with 1 μL of NaOH 0.5 M (final concentration 5 mM); after 30 min, the reaction mixture was diluted with 400 μL of PB 50 mM pH 7.4 to quench the reaction. The obtained 200 μM solution of H_2_DCF in ethanol/PB (1:5) was used for the experiments. The DCF assay was performed on a multiwell plate using a CLARIOStar plate reader. The fluorescent emission of DCF was measured at 530 nm upon excitation at 485 nm. H_2_DCF (10 μM final concentration) was added to a solution of Cu^II^/Cu^II^-TSC in HEPES 50 mM pH 7.4; then H_2_O_2_ and GSH were added to initiate the reaction.

### HPLC

HPLC analysis GSSG formation was performed using a Hitachi Primaide instrument equipped with a C18 column (Xbridge Peptide BEH C18 column from Waters, 4.6 mm × 150 mm, pore size 300 Å, particle size 3.5 μm), using 0.1% aqueous TFA (solvent A) and 90% CH3CN/0.1% TFA in water (solvent B) with a linear gradient from 5% to 10% solvent B in 7 min.

### Low-Temperature Luminescence

Low-temperature luminescence spectra were recorded using a FluoroMax Plus spectrofluorometer (Horiba Scientific) equipped with a cylindrical quartz dewar filled with liquid nitrogen (at 77 K). 500 μL samples were transferred to quartz tubes with 4 mm inner diameter and freeze-quenched into liquid nitrogen before their introduction in the dewar. Emission spectra were obtained with excitation at 310 nm.

### EPR Spin Scavenging

EPR spin scavenging experiments were performed at room temperature (*T* = 295 ± 1 K) using an EMX-plus (Bruker Biospin GmbH, Germany) X-band EPR spectrometer equipped with a high sensitivity resonator (4119HS-W1, Bruker). The g factor was calibrated in the experimental conditions using the Bruker strong pitch (*g* = 2.0028). Samples were introduced into glass capillaries (Hirschmann, 25 μL) sealed at both the ends and rapidly transferred into the EPR cavity for measurement. The principal experimental parameters were microwave frequency of ∼9.85 GHz, microwave power of ∼4.5 mW, modulation amplitude of 1 G, time constant of ∼5 ms, and conversion time of ∼12.5 ms. Every 17 s, a single scan (sweeping time of ∼10 s) was then acquired to obtain the kinetics of TEMPOL reduction over ∼60 min. All spectra were best simulated, and the resulting simulations were doubly integrated to relatively quantify the concentration of remaining TEMPOL. Data analysis and simulations based on experimental data were performed using Xenon (Bruker Biospin GmbH, Germany) and lab-made routines based on Easyspin Toolbox under Matlab (Mathworks) environment.^22^

### UV–Vis Spectroscopy

UV–vis measurements were carried out in 1 cm path quartz cuvettes using an Agilent Cary 60 spectrophotometer.

### DFT

The Gaussian16 software package^23^ was used to carry out all the calculations in the framework of DFT and its TD-DFT extension. The hybrid meta functional used for geometry optimizations and frequency calculations is M05.^24^ Such a functional was adopted because it was demonstrated to be able to accurately model metal-containing compounds interactions.^25^ Frequency calculations were carried out to both confirm the nature of minima and transition states, number of imaginary frequencies 0 or 1, respectively of the optimized structures and to calculate zero-point energy (ZPE) corrections. The standard 6-311G* basis set of Pople was employed for Cu, C, N, O, H atoms together with the 6-311+G* basis set for S atoms. In order to simulate the water physiological environment and the impact of solvation on the reactivity, the solvation model based on density, SMD, was adopted for all geometry optimizations, because it can be consistently used for any charged or uncharged solute in any solvent or liquid medium.^26^ Aiming at containing the computational costs and to properly simulate a high thiol concentration reproducing the experimental conditions, cysteine was used instead of GSH to explore the reaction mechanism. Relative Gibbs free energies (Δ*G*), including thermal corrections at 298.15 K, were calculated for all the located stationary points of the path with respect the sum of the free energies of separated reactants fixed as the zero reference energy of the system. Grimme dispersion corrections were included using the atom pairwise additive scheme,^27^ DFT-D3 method, to properly take into account the contribution of weak interactions. Molecular electrostatic potential (MEP) calculations were carried out for some key species using the Gaussian 16 program.^23^ The hardness, η, values have been calculated adopting the formula η = (*I* – *A*)/2 where *I* is the ionization potential defined by the difference *E*^*N*–1^ – *E*^*N*^ and *A* is the electronic affinity defined by the difference *E*^*N*^ – *E*^*N*+1^ for the systems with *N*, *N* – 1, and *N* + 1 electrons.

## Supporting Information Available

EPR spin scavenging measurements. UV–vis spectrum of the GS-bound Cu^I^ in the presence of Dp44mT upon air exposure. Optimized geometrical structures of the stationary points intercepted along the Cu^II^-3AP complex reduction pathway. Schematic representation of the stationary points intercepted along the Cu^II^-Dp44mT complex reduction pathway. Intercepted stationary points together with their relative energies describing the imidazole detachment from the Cu center of the two Cu^II^-3AP and Cu^II^-Dp44mT complexes. Effect of imidazole and glycine on Cu^II^-Dp44mT and Cu^II^-3AP complexes reduction by GSH monitored over time by UV–vis spectroscopy (PDF)
