# Supplementary material for: Dual Role of Glutathione as a Reducing Agent and Cu-Ligand Governs the ROS Production by Anticancer Cu-Thiosemicarbazone Complexes
Source: Inorg Chem. 2023 Feb 20;62(9):3957–64. doi: 10.1021/acs.inorgchem.2c04392 (PMC9996813; doi:10.1021/acs.inorgchem.2c04392)
Supplement: Supplementary file 1 — ic2c04392_si_001.pdf [file ic2c04392_si_001.pdf]

Supporting Information

Dual Role of Glutathione as a Reducing Agent and Cu-Ligand Governs the ROS Production by Anticancer Cu-Thiosemicarbazone Complexes

Alessandra G. Ritacca,[a]# Enrico Falcone,[b]# Iman Doumi,[b] Bertrand Vileno,[b] Peter Faller,[b,c]* Emilia Sicilia[a]*

[a] Department of Chemistry and Chemical Technologies, Università della Calabria, Ponte P. Bucci, 87036 Arcavacata di Rende (CS) (Italy).

[b] Institut de Chimie (UMR 7177), University of Strasbourg – CNRS, 4 Rue Blaise Pascal, 67081 Strasbourg, France.

[c] Institut Universitaire de France (IUF), 1 rue Descartes, 75231 Paris, France

#These authors contributed equally.

*corresponding authors: emilia.sicilia@unical.it (ES); pfaller@unistra.fr (PF)

Contents

Figure S1. EPR spin scavenging measurements S2

Figure S2. UV-vis spectrum of the GS-bound Cu^{I} in the presence of Dp44mT upon air exposure S2

Figure S3. Fully optimized geometrical structures of the intercepted stationary points along the reduction pathway of the Cu-3AP complex. S3

Figure S4. Schematic representation of the intercepted stationary points along the reduction pathway of the Cu^{II}-Dp44mT complex. S4

Figure S5. Intercepted minima and transition states, together with their relative energies (kcal/mol), describing the Imidazole detachment from the Cu center of the two Cu^{II}-3AP and Cu^{II}-Dp44mT complexes. S5

Figure S6. Effect of imidazole and glycine on Cu^{II}-Dp44mT complex reduction by GSH in the presence of monitored over time by UV-Vis spectroscopy. S6

Figure S7. Effect of imidazole and glycine on Cu^{II}-3AP complex reduction by GSH monitored over time by UV-Vis spectroscopy. S7

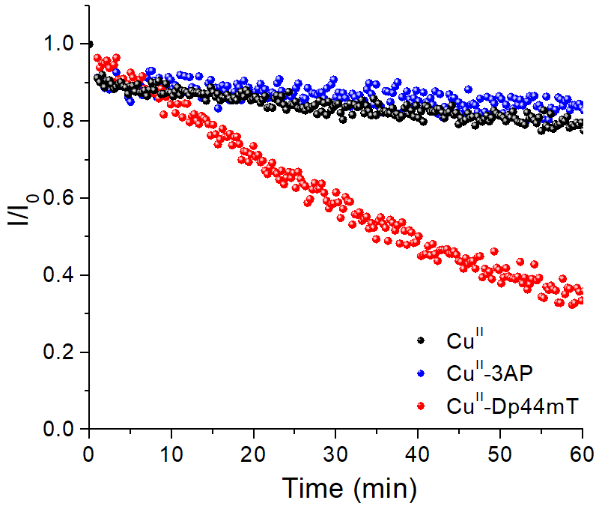

Figure S1. Decay of TEMPOL EPR signal in the presence of Cu^{II} (black dots), Cu^{II}-3AP (blue dots) or Cu^{II}-Dp44mT (red dots) and GSH. Conditions: [Cu^{II}] = 27 µM, [TSC] = 30 µM, [GSH] = 3 mM, [TEMPOL] = 20 µM, HEPES buffer 100 mM pH 7.4.

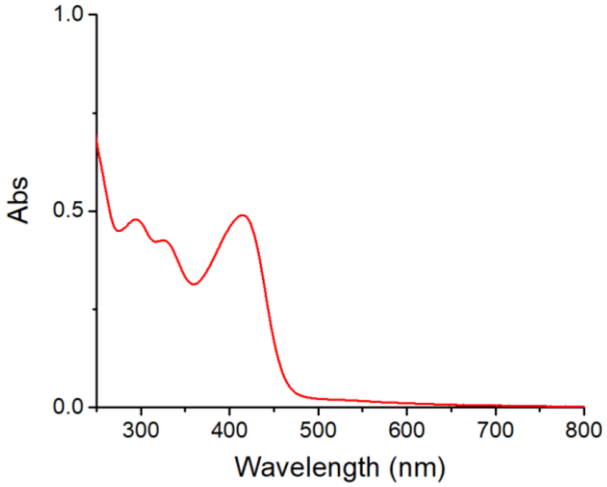

Figure S2. UV-vis spectrum of the GS-bound Cu^{I} in the presence of Dp44mT upon air exposure. Charge transfer bands at 327 nm and 415 nm reveal the formation of a ternary (GS^{-})-Cu^{II}-Dp44mT adduct. Conditions: [GSH] = 3 mM, [$Cu^{I}(MeCN)_4PF_6$] = 27 µM, [TSC] = 30 µM, HEPES 100 mM pH 7.4 (DMSO, MeCN < 1%).

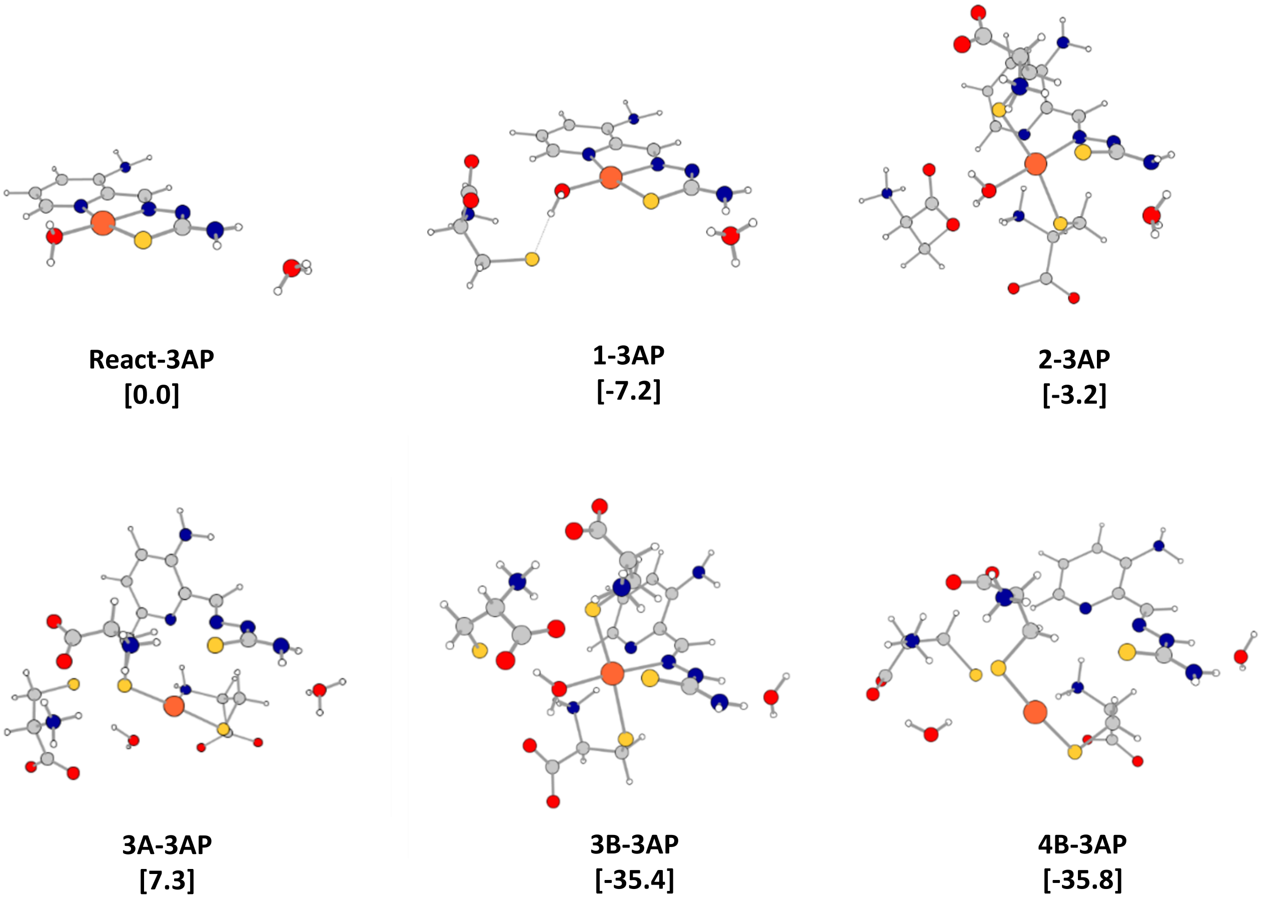

Figure S3. Fully optimized geometrical structures of the intercepted stationary points describing the mechanism of the reaction between the Cu^{II}-3AP complex and deprotonated cysteine. Relative energies are in kcal/mol

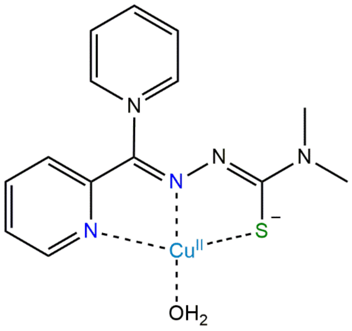

React-Dp44mT
[0.0]

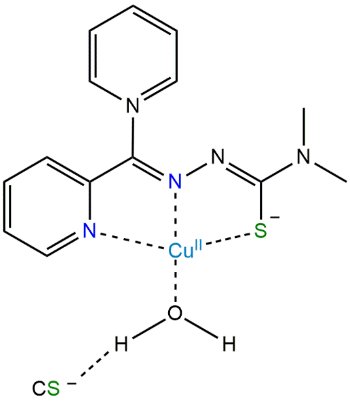

1-Dp44mT
[-10.4]

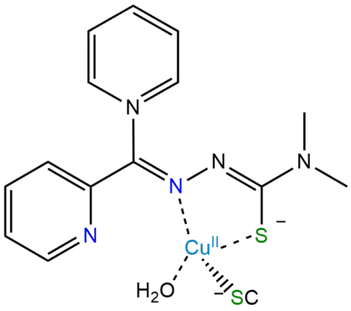

2-Dp44mT
[-11.7]

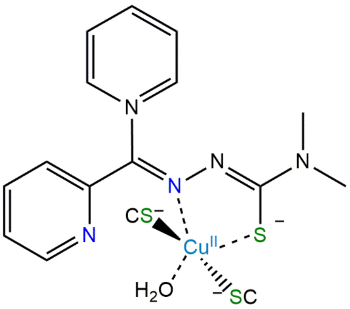

3-Dp44mT
[-4.7]

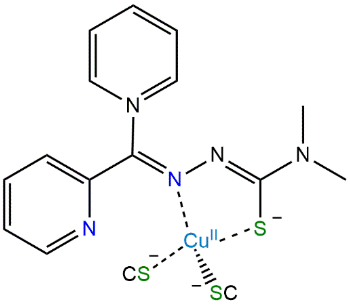

4-Dp44mT
[-6.4]

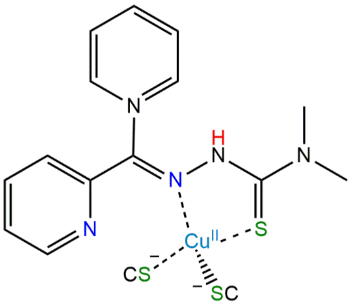

5-Dp44mT
[-41.4]

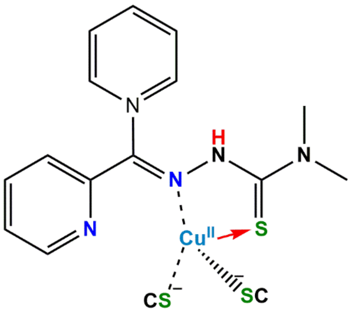

TS6-Dp44mT
[-39.4]

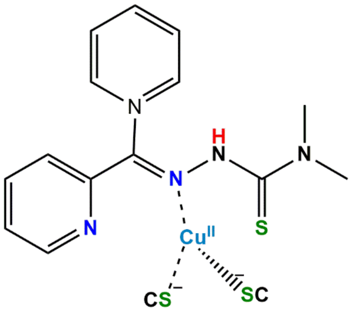

7-Dp44mT
[-41.2]

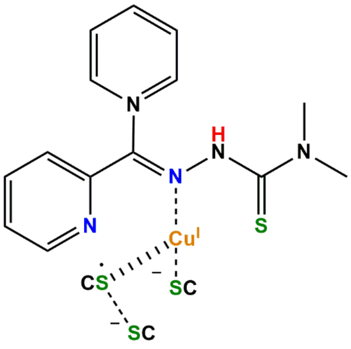

8-Dp44mT
[-36.4]

Figure S4. Schematic representation of the intermediates of the reaction between Cu^{II}-Dp44mT and deprotonated cysteine. Relative energies are in kcal/mol.

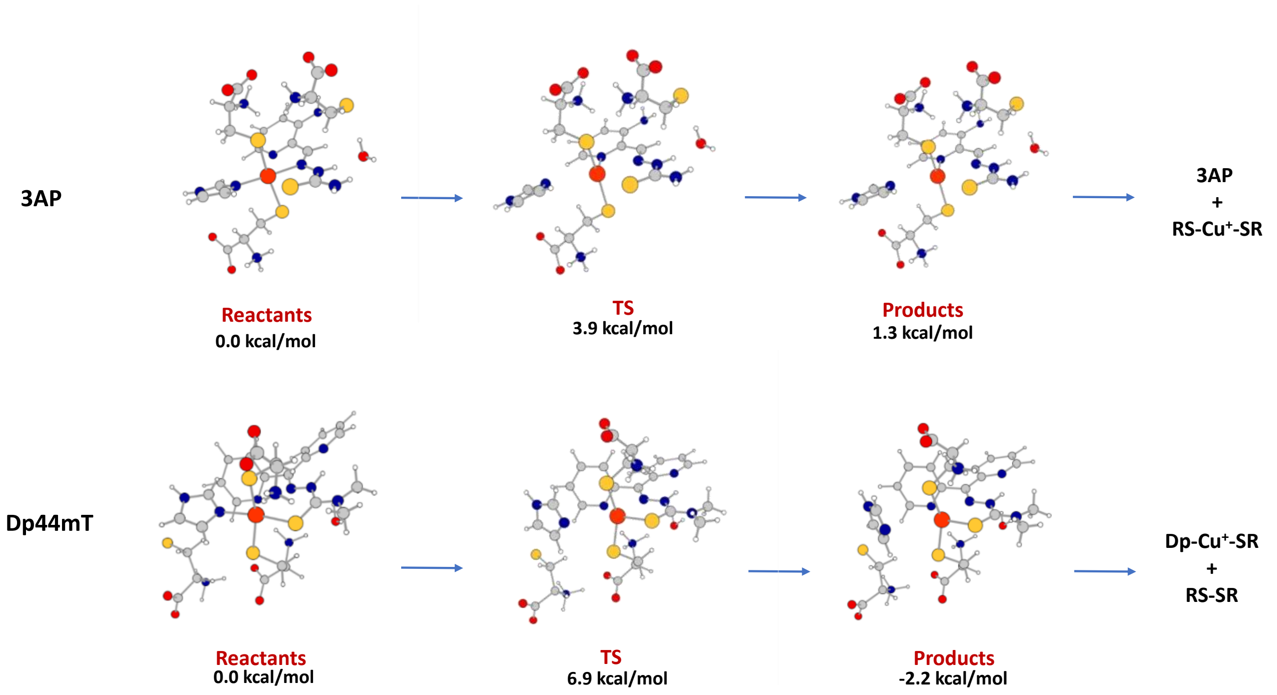

Figure S5. Pathways, that include intercepted minima and transition states, describing the Imidazole detachment from the Cu center of the two Cu^{II}-3AP and Cu^{II}-Dp44mT complexes together with their relative energies (kcal/mol),

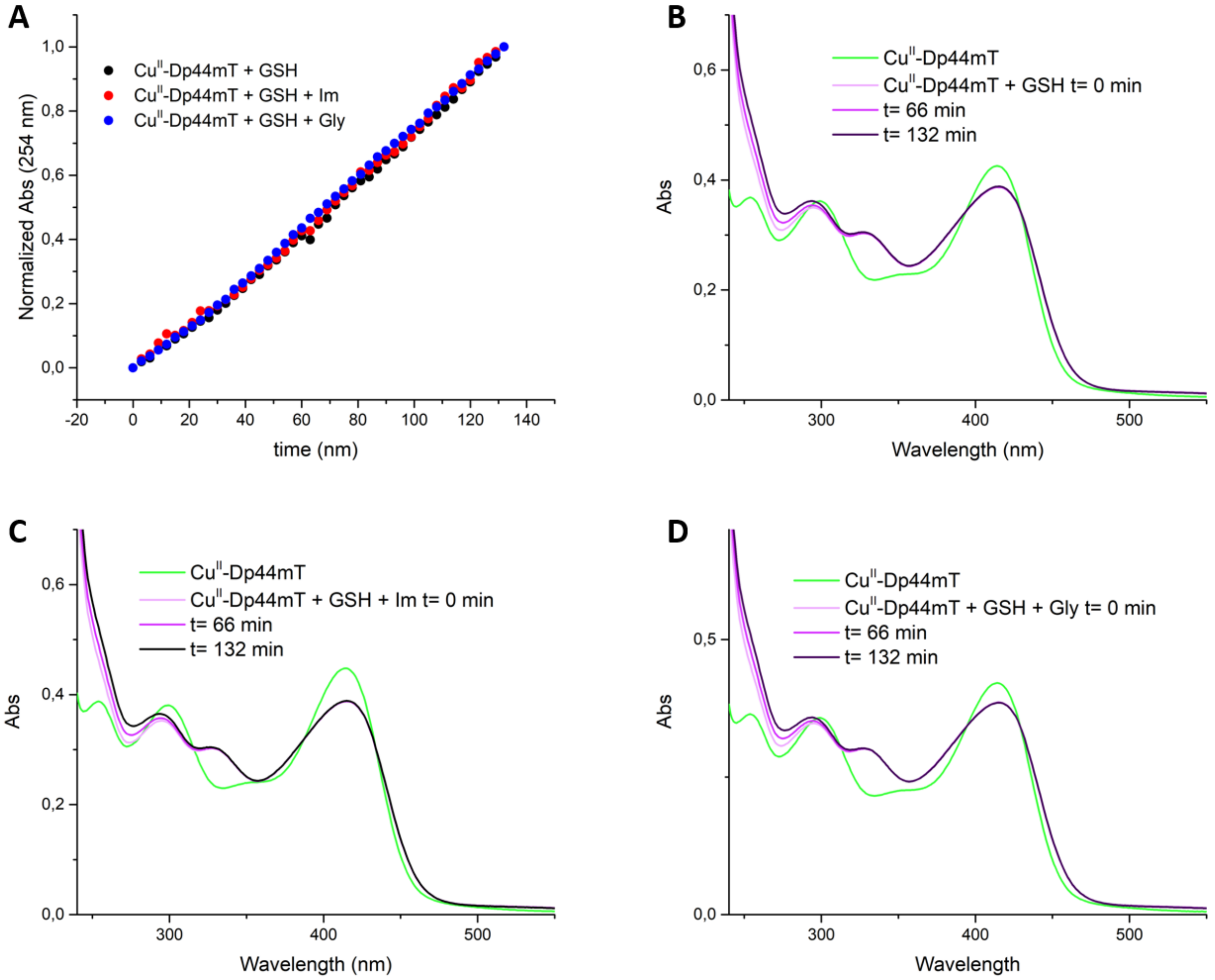

Figure S6. Reaction of the Cu^{II}-Dp44mT complex with GSH in the presence of imidazole (Im) or glycine (Gly) monitored over time by UV-vis absorbance spectroscopy. (A) Time-dependent increase in absorbance at λ_{max} (254 nm) of the GSSG band in the absence (black) and presence of Im (red) or Gly (blue) . Intermediate spectra were collected at 3 min intervals. (B-D) UV-Vis spectra at given time points of the reaction of Cu^{II}-Dp44mT with GSH (B), GSH and Im (C), GSH and Gly (D). Experimental conditions: [Dp44mT] = 30 µM, [Cu^{II}] = 27 µM (ratio Dp44mT:Cu^{II}, 1:0.9), [GSH] = 3 mM, [Im] = 3 mM, [Gly] = 3 mM, 100 mM HEPES buffer, pH 7.4.

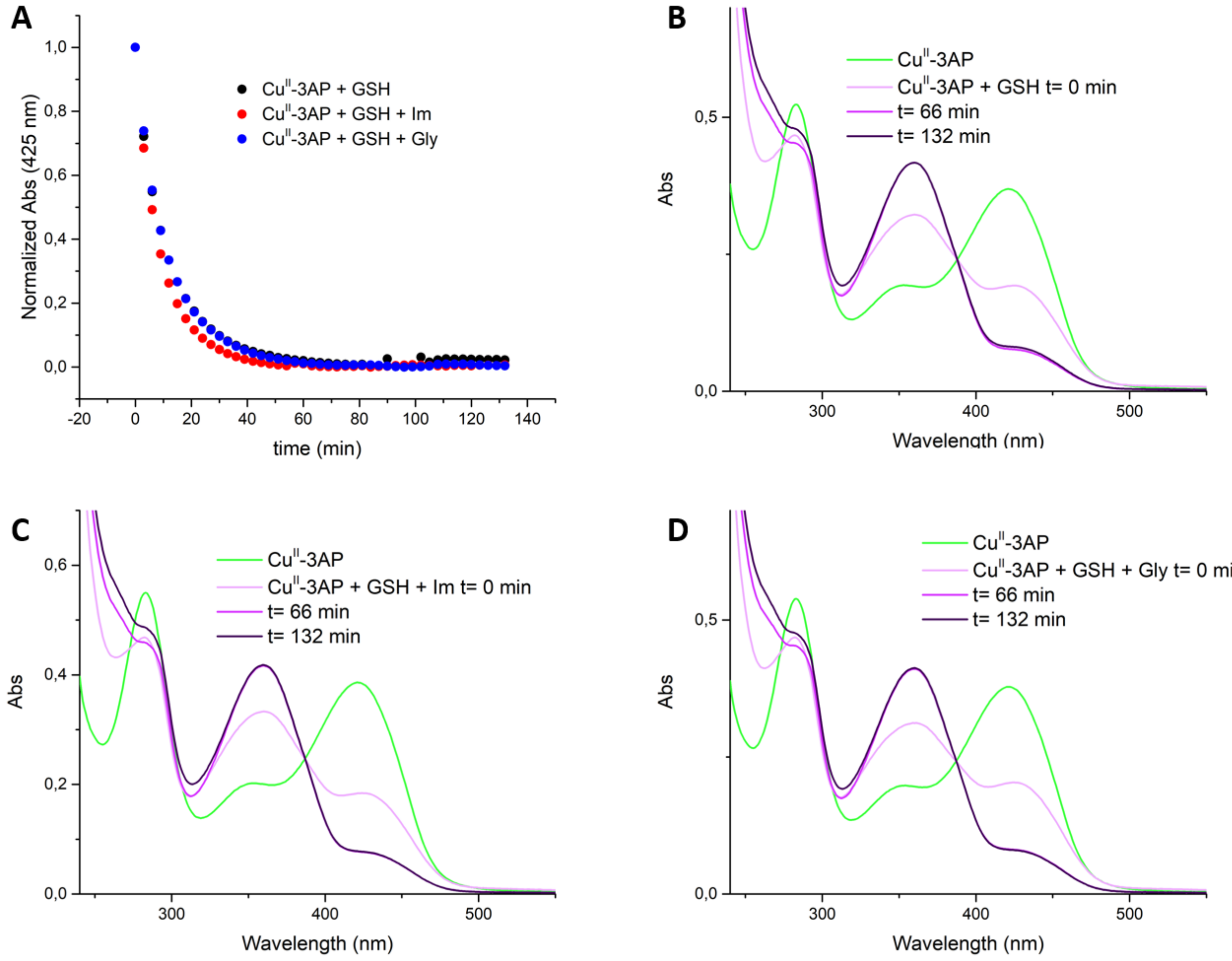

Figure S7. Reaction of the Cu^{II}-3AP complex with GSH in the presence of imidazole (Im) or glycine (Gly) monitored over time by UV-vis absorbance spectroscopy. (A) Time-dependent reduction of Cu^{II}-3AP monitored by the absorbance decrease at the λ_{max} (425 nm) of the CT band of the GS-Cu^{II}-3AP ternary adduct in the absence (black) and presence of Im (red) or Gly (blue). Intermediate spectra were collected at 3 min intervals. (B-D) UV-Vis spectra at certain time points during the reaction of Cu^{II}-3AP with GSH (B), GSH and Im (C), GSH and Gly (D). Experimental conditions: [3AP] = 30 μM , [Cu^{II}] = 27 μM (ratio Dp44mT:Cu^{II}, 1:0.9), [GSH] = 3 mM, [Im] = 3 mM, [Gly] = 3 mM, 100 mM HEPES buffer, pH 7.4..
